# Generation of Tactile Maps for Artificial Skin

**DOI:** 10.1371/journal.pone.0026561

**Published:** 2011-11-10

**Authors:** Simon McGregor, Daniel Polani, Kerstin Dautenhahn

**Affiliations:** University of Hertfordshire, Hatfield, Herts, United Kingdom; Indiana University, United States of America

## Abstract

Prior research has shown that representations of retinal surfaces can be learned from the intrinsic structure of visual sensory data in neural simulations, in robots, as well as by animals. Furthermore, representations of cochlear (frequency) surfaces can be learned from auditory data in neural simulations. Advances in hardware technology have allowed the development of artificial skin for robots, realising a new sensory modality which differs in important respects from vision and audition in its sensorimotor characteristics. This provides an opportunity to further investigate ordered sensory map formation using computational tools. We show that it is possible to learn representations of non-trivial tactile surfaces, which require topologically and geometrically involved three-dimensional embeddings. Our method automatically constructs a somatotopic map corresponding to the configuration of tactile sensors on a rigid body, using only intrinsic properties of the tactile data. The additional complexities involved in processing the tactile modality require the development of a novel multi-dimensional scaling algorithm. This algorithm, ANISOMAP, extends previous methods and outperforms them, producing high-quality reconstructions of tactile surfaces in both simulation and hardware tests. In addition, the reconstruction turns out to be robust to unanticipated hardware failure.

## Introduction

Spatial projections of various sensory (and motor) surfaces onto neural structures are common in neuroanatomy, where they are known as *topographic maps*. For instance, in the primary visual cortex (V1), neighbouring cells in the retina activate neighbouring cortical columns (*retinotopy*). In the auditory system, similar frequency components of sound activate neighbouring cells in the organ of Corti, and project to neighbouring locations on the cortex (*tonotopy*).

The developmental mechanisms which allow topographic maps to form in animals are complex and not completely understood, but in many animals, the neurological development of sensory systems are known to depend on both prenatal and antenatal sensory stimulation. For instance, the development of visual depth perception in cats depends on active participation in visual experience [1]. Sensoritopic map formation involves self-organising processes which are guided by sensory signals: ferrets can develop retinotopic maps on the auditory cortex, if their visual afferent nerves are surgically rerouted to their auditory cortex [2]; in mice, spontaneous *in utero* waves of activation on the retina are involved in topographic map formation [3]. Simulations of retinotopic map formation based on *self-organising maps* have been claimed to accurately model the visual cortex, including reproducing features such as ocular dominance maps and visual after-effects [4]. Self-organising maps have also been used to model tonotopic features of the auditory cortex in certain bats [5].

The basis for the success of these structural mappings derives from the fact that signals provided to the brain by the sensory organs have statistical properties which reflect the structure of those organs and the environment. In particular, it suggests that there is sufficient *intrinsic* information in these sensory signals to allow the brain to reconstruct a significant part of the organism's sensory topology. Our aim in the present paper is to apply this principle to the development of algorithms for robotics.

An ideal flexible robot ‘brain’ would build a model of its sensorimotor contingencies *ab initio* and *in situ*, with the algorithm making only minimal assumptions about the robot's sensors, actuators and environment. Suitable approaches to robot learning of sensorimotor contingencies include explicit approaches such as the *uninterpreted sensors and actuators* paradigm (introduced in [6]), dimensionality reduction and estimation [7], the information structure approach [8] and model-building work such as [9]; the possibility of implicit (as opposed to explicit) sensorimotor contingency learning has been explored in work such as [10], which uses coupled chaotic systems to explore stable sensorimotor patterns. This perspective, whether the robot learns using an explicit learning algorithm or using emergent dynamics, is purely intrinsic to the robot in that it uses only the robot's sensors in a very general environment. This stands in contrast to alternative approaches (see, e.g. [11]) which use a specific externally defined calibration environment to initialise a robot's sensory model.

The relevance of intrinsic approaches derives from enactive and embodied/situated perspectives on cognition. These argue that the whole process of cognitive perception and action is essentially driven by the specific constraints determining the sensorimotor contingencies which can be learned intrinsically by an organism; see, for instance, [7].

The uninterpreted sensor approach in robotics from [6], referred to above, has been developed further [12], [13]. generalised the original approach by using a metric from information theory (the *information distance*). This allowed the visual field of an AIBO robot to be reconstructed using only raw data from the camera pixels in a “model-free” manner, making no further assumptions about the robot's sensors or environment. In another study [14], described a refined method for sensory reconstruction using Gaussian process assumptions and a sparse linear algebra approximation technique. This allowed the reconstruction of a complex sensor geometry (the Stanford bunny) in simulation and a visual field in recorded robot data.

This article describes elaboration and application of these methods to the reconstruction of a surface embedded in 3D from intrinsic tactile sensor data: a *somatotopic map*. To the authors' knowledge, this is the first time such a reconstruction has been performed on simulated or real hardware data. The simulated sensor geometry reconstructed in [14] is similar in some ways to our tactile surfaces (in that it is embedded in 3D), but does not model a physical tactile surface: sensors were stimulated by multiple point source pulses, with the response at each sensor depending on the Euclidean distances of the sensor from the point sources. In this scenario, the 2D manifold on which they lie lacked any special meaning in the simulation since there was no simulated solid body affecting the sensory input; the sensors were more like radio receivers situated in empty space. Somatotopic maps also differ from the retinotopic reconstructions studied so far (such as [13], [14]) in two crucial respects. Firstly, the tactile modality provides far sparser data than the visual modality (most of the signals essentially vanish most of the time), and involves identifying quite different features of the world (surfaces directly in contact with the organism). Secondly, tactile sensory surfaces are harder to reconstruct than visual ones: locally, the topology of the skin surface is two-dimensional, but it is much more likely than the visual field to incorporate topologically more involved features such as holes, which require a three-dimensional Euclidean embedding. In this respect, tactile surfaces are quite unlike visual or auditory surfaces.

To address the particular challenges posed by the sensoritopic scenario, we introduce introduce a new algorithm ANISOMAP, constituting a generalisation of the well-known ISOMAP algorithm [15]. This method is based on *graph geodesics*, first constructing a mathematical graph of nodes (representing sensors) connected by weighted edges ( which in our case represent statistical dissimilarities), then using a well-known algorithm to find the shortest path lengths between each pair of sensors. The corresponding graph distances can be used as input to another well-known algorithm to produce a sensoritopic reconstruction. The procedure is described in detail in the “Methods” section.

Somatotopic map formation has previously been modelled using Self- Organising M aps (SOMs) (e.g. [16], [17]). Interestingly, although SOM methods exist for addressing the problems we have mentioned that arise from non-trivial topologies [18], [19], to our knowledge they have not yet been applied to somatotopic maps. The SOM approach differs from that used in [13] and extended in this paper: our method (developed in the context of robot control) models each individual sensor as a single point in the map; by contrast, SOMs are maps of holistic sensory vectors, and are highly un likely to have a one-to-one relation between sensors and map neurons. Moreover, the space represented by a SOM map corresponds to a discrete topology on the neurons themselves; our approach allows for coordinate variables to be coded directly. While SOMs are probably more biologically realistic, our approach can reconstruct a three-dimensional tactile surface using only a relatively small number of reconstruction points.

## Results

We compared the ANISOMAP technique to other methods (information distance and regular ISOMAP), and to a null hypothesis, in the reconstruction of tactile surfaces from sensor data:

- Simulated tactile data from sensors on several differently shaped rigid bodies (sphere, cylinder, plane and Y-shape) in two different physical simulation scenarios (one involving bombardment by small balls; one involving rolling along a landscape).
- Actual tactile data recorded from a hexagonal patch of prototype artifical skin in the lab.

Details of the procedures used to generate the data, and of the reconstruction techniques, are given in the “Methods” section. Note that one of the sensors in the physical prototype failed between experimental runs; data from this sensor was nevertheless included in the input to the reconstruction methods for all hardware experiments, allowing us to observe the performance of the algorithms in the face of hardware failure.

In the ideal reconstruction, the distances between each pair of reconstructed sensor positions would be identical to the original (simulated or physical) distances between that pair of sensors (up to a multiplicative constant). Hence, the statistical correlation between reconstruction distance and original distance (considered over all pairs of sensors in a particular object) was chosen as a quantitative measure of reconstruction quality. We refer to this measure as “distance-correlation”. Although data from the failed hardware sensor was allowed to affect the reconstructions, its position in the reconstructed geometry was excluded from the calculation of reconstruction quality.

Note that some care must be taken in interpreting a distance-correlation value. Although a distance-correlation is a standard Pearson's 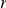 value ranging between 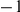 and 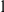, it will not necessarily have an expected value of zero in the absence of meaningful relations between original geometry and reconstructed geometry. This is because the metric embedding of both original and reconstructed geometry poses a similar systematic constraint on the entire matrix of pairwise distances (for instance, the distance from a point to itself will always be zero). Randomisation of data is often used to distinguish significant effects from a baseline; see for instance the robust mutual information measure in [20]. In an analogous fashion, we compare the algorithms' performance to a randomised baseline, to reduce any systematic bias in our performance measure. The randomised baseline is computed by taking the reconstructions provided by the algorithms and randomly permuting the sensor labels attached to points in the reconstruction. This provides a reasonably conservative estimate of how much of the distance-correlation score is attributable to artefacts of non-independence in metric matrices. In this way, we ask the question: how much of the algorithms' performance can be attributed in principle to the “gross” reconstructed geometry they produce (ignoring where they locate each sensor within that geometry), and how much can be attributed to the specific map between sensors and points?

**Figure 1:**
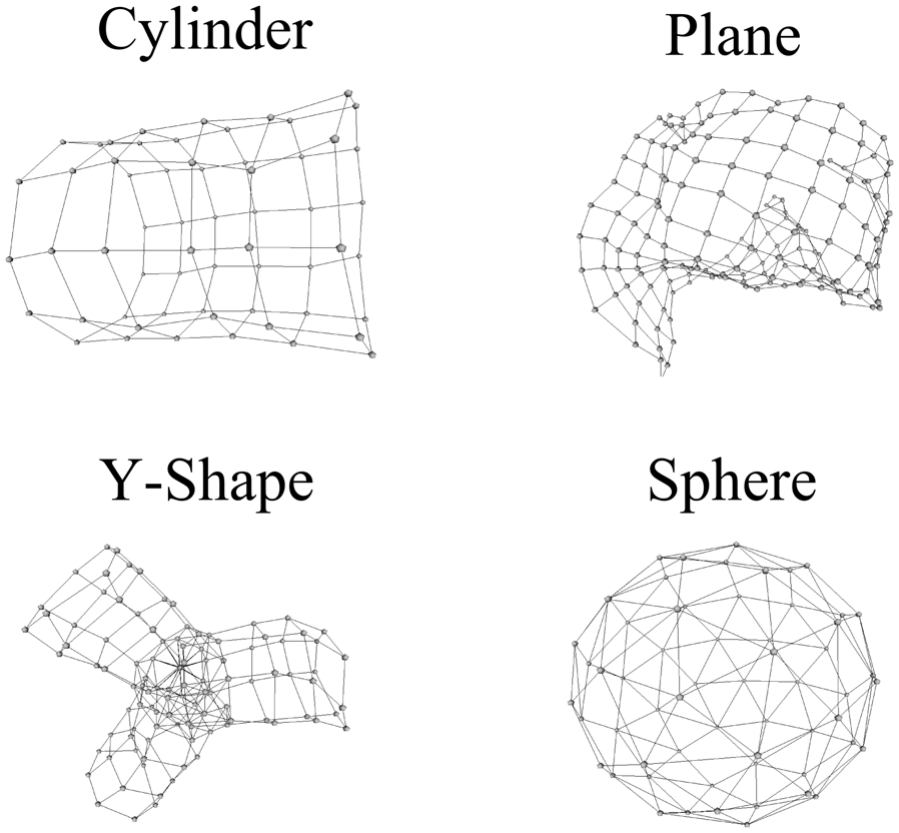
Typical reconstructions for 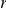-ANISOMAP: cylinder, plane, y-shape and sphere.

**Figure 2:**
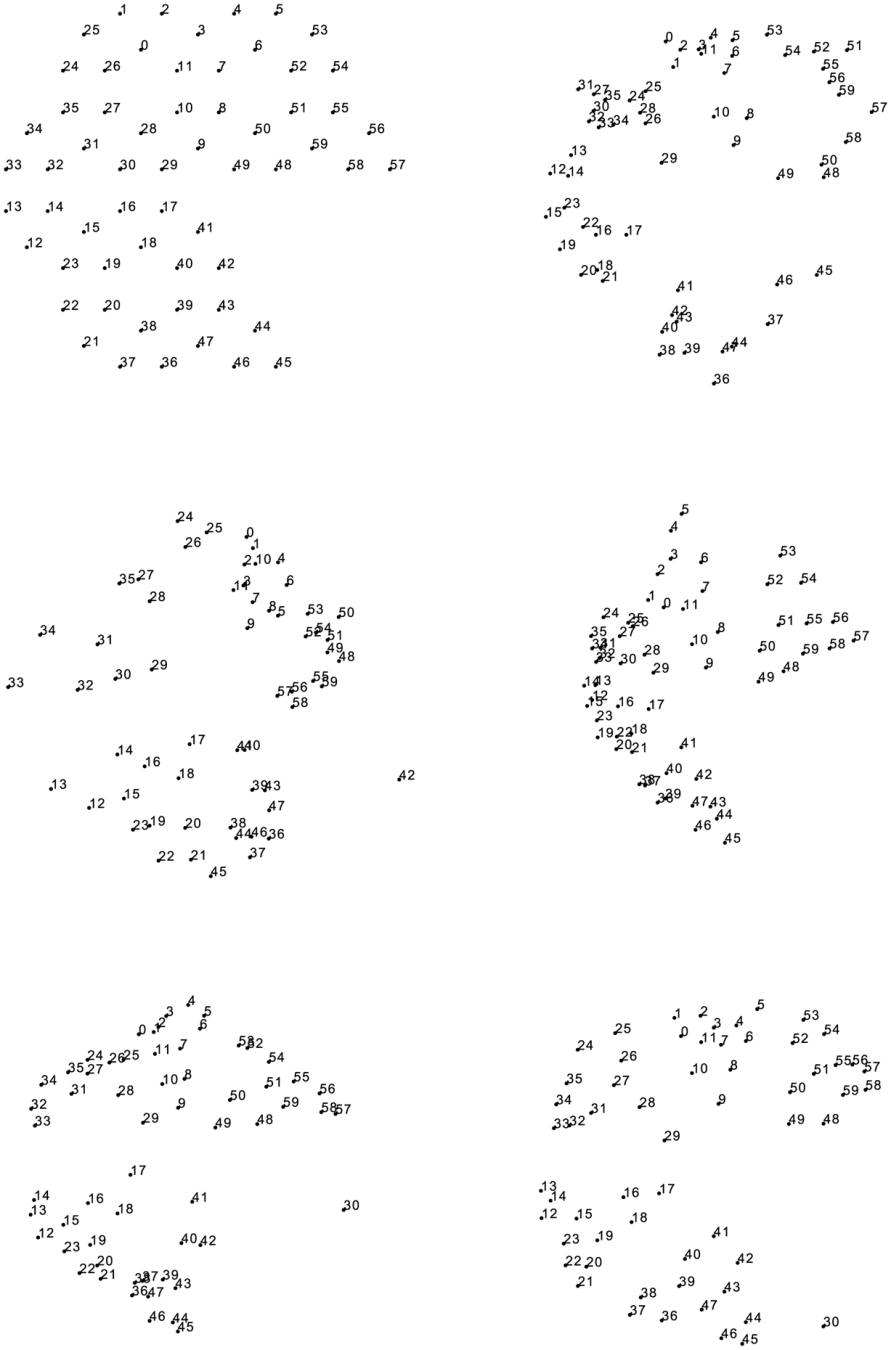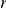-ANISOMAP reconstructions for the hardware experiments. Top left: actual configuration; sensor 30 failed after experiment 3.

Over all the reconstruction scenarios, the 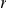-ANISOMAP algorithm was consistently among the best performing according to our performance measure. Fig. 1 shows typical (randomly chosen) reconstructions by 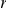-ANISOMAP for the bombardment scenario. Fig. 2 shows the 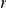-ANISOMAP reconstructions for each hardware experiment. Note that the faulty sensor 30 is isolated from the others by the 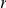-ANISOMAP algorithm in Fig. 2. Figures S1, S2, S3, S4 in supporting information online show typical (randomly selected) reconstructions of the sphere, Y-shape, plane and cylinder geometries for each reconstruction method, and Figures S5, S6, S7, S8, S9 show all hardware reconstructions.

**Figure 3:**
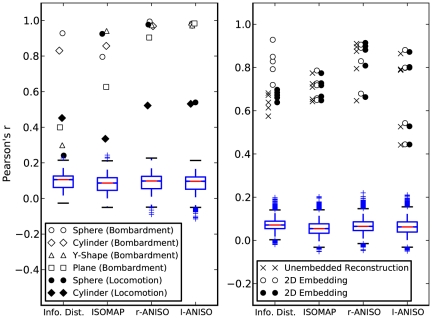
Reconstruction quality for 4 algorithms on simulation and hardware data. Boxplots show reconstruction quality (correlation of reconstructed sensor distance with original distance) of 10,000 random permutations of the algorithm's output to compare to null hypothesis. Small ‘+’ symbols are boxplot outliers (outlier distance is 1.5 times the inter-quartile range).

Fig. 3 shows the performance of the candidate algorithms in simulation and in hardware, measuring correlations between reconstructed sensor distances and original sensor distances. Resampling tests (corresponding to boxplots at the bottom of the graphs) show unambiguously that all algorithms perform far better than random guessing when assigning sensors to points in the reconstructed geometry (in terms of distance correlation).

In applying qualitative judgement to these figures, we suggest that high quantitative distance correlation corresponds to a reconstruction which respects the topological and usually geometric features of the original.

## Discussion

We have shown that it is possible to approximately reconstruct the topology and geometry of skin in 2 or 3 dimensions based only on intrinsic data from tactile sensors. These reconstructions were achieved both in simulation and in hardware. We conclude that tactile sensory data, in the context studied by our experiments, contains significant implicit information about the sensory interface between an agent and its environment - enough to allow the spatial structure of that interface to be retrieved. This information does not need to be “engineered in”; it is a natural consequence of the agent's embodiment. In this respect, we extend previous results in e.g. [6], [13] on kinaesthetic and visual data to a novel sensory modality.

Interestingly, successful reconstruction in 3 dimensions required a novel algorithmic technique we call ANISOMAP. While the information distance method used in [13] performed well in 2 dimensions, the algorithm relies heavily on 2D embedding. The reasons for this have been explored in [21]: essentially, the information distance method has a tendency to prefer assigning sensor positions to the surface of a hypersphere. The ratio of available surface to volume of a 2-sphere is small enough for this distortion to be relatively minor; however, for a 3-sphere the distortion is significantly more pronounced. This should not be seen as a reflection on information-theoretic methods in general; they still offer better theoretical application to cases where assumptions of linear correlation do not hold. For instance, if two sensors measure the same quantity, but one is linear and the other is a radial basis function around an optimal response point, the information-theoretic model is more likely to identify the relation between the sensors. This “sphericisation” problem stems in part from the fact that all statistically independent pairs of sensors are essentially assigned the same distance from one another. However, statistical *in*dependence tells us relatively little about the sensoritopic relationship between sensors; the further apart sensors are, the less information their joint statistics provide about their sensory distance. In contrast to the information distance approach, which assigns a finite (and approximately fixed) distance to independent pairs of sensors, we address this problem by assigning an arbitrarily large dissimilarity to independent pairs of sensors (using reciprocation). Our novel ANISOMAP algorithm then effectively refines over-estimated long distances downwards based on chains of shorter (more reliable) distances. No specific threshold defines what constitutes “long” or “short”; the principle operates uniformly at all scales.

**Figure 4:**
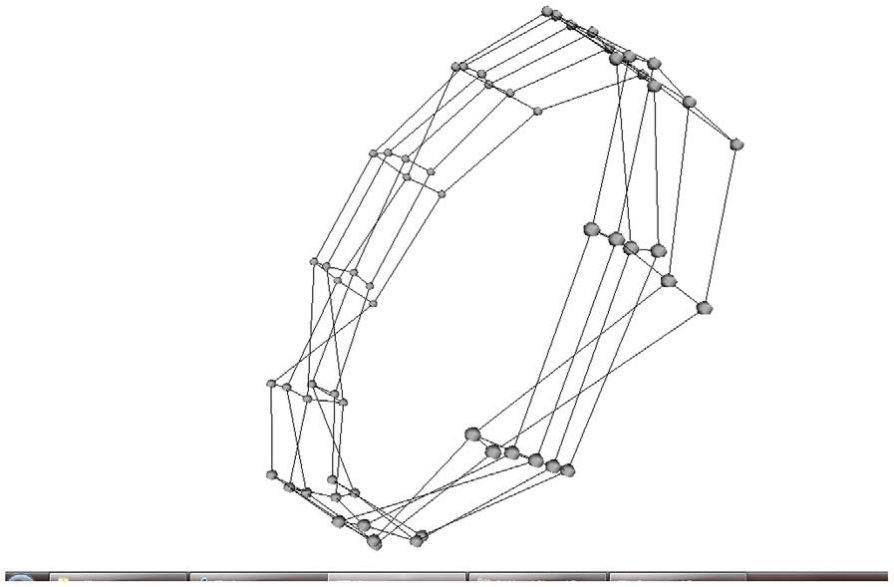
Cylinder locomotion reconstruction. Typical reconstruction of the cylinder in the locomotion scenario, showing axial compression.

The ANISOMAP methods produced high correlation between reconstructed and original distances in nearly every case. The exception was for the cylinder geometry in the locomotion scenario, where the numerical correlations were relatively poor for all reconstruction methods. In this scenario the most statistically similar sensors lie on axial lines on the tactile surface, because these lines typically form the contact points between a cylinder and the ground. Consequently, the reconstructions tended to place points on these lines closer to each other, relative to their neighbours on neighbouring axial lines. The results were reconstructed cylinders which reconstructed the topology of the original sensor layout reasonably well, but were compressed in the axial direction compared to the original geometry (see Fig. 4). This is a particular illustration of the fact that a sensoritopic map will often closely match the sensory surface's geometry, but sometimes has a different structure, reflecting the wider sensorimotor properties of a particular embodiment.

Studying the intrinsic information content of sensory data is important for the understanding of embodied cognition in nature. Although we do not claim that the algorithm we use here is biologically realistic, it allows us to demonstrate that artificial tactile sensory data contains information about more than just the immediate tactile environment: it also inherently carries information about the structure of the tactile sensory surface. When the same relations apply in biology, we can conjecture that organisms might exploit this fact during development, using a different mechanism. To test this, ANISOMAP or similar artificial reconstruction techniques could be applied to recorded neural data, to establish that such informational relations hold.

Using the quantitative measure, across all hardware and simulation experiments, the best 3D reconstruction was invariably one of the ANISOMAP variants. Since the 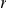-ANISOMAP algorithm performed reasonably or excellently in all cases (both qualitatively and quantitatively), including responding appropriately to unexpected hardware failure, our conclusion from the experiments is to propose this method as a promising candidate for developing further approaches to sensoritopic reconstruction and automatic self-calibration in robotics. We expect that that artificial skin sensors will provide enough intrinsic information to allow reasonably accurate reconstructions of more complex somatosensory surfaces than the ones we studied; this observation will feed into the debate about why somatotopic maps in the cortex are not faithful to the topology of the skin [17], [22], [23].

In conclusion, like visual and auditory surfaces, and despite being more complex in their topology, immediate tactile sensory signals in artificial skin inherently contain information about the tactile surface. This is due to local spatial relations between pairs of tactile sensors being reflected in their joint statistics. The information contained in these pairwise statistical relationships can be integrated globally by a novel algorithm, ANISOMAP, and be shown to capture most of the spatial structure of the tactile surface.

## Methods

This section describes the methods used in sensoritopic reconstruction from three distance estimate methods (information distance, ISOMAP and ANISOMAP). It also briefly describes the scenarios and geometries used in the simulation experiments, and the parameters of the physical hardware experiments. Further details and raw data are provided in supplementary information online.

The methods we used to estimate sensory distance (information distance, ISOMAP and ANISOMAP) are described in detail below.

We begin with an overview of our experimental methodology in general terms. The two sections after the overview deal with the collection of artificial tactile data - the first section addressing artificial skin simulation and the second section describing lab experiments on artificial skin hardware.

### General Methodology

Here we give an overview of our experimental process from the measurement of tactile data to the generation of sensoritopic reconstructions. Later sections describe in more detail how the tactile data was obtained and and how the reconstruction algorithms operated.

Following [13], we considered sensoritopic reconstruction methods based on statistical distance estimation. Each pair of sensors was assigned a distance based on the joint statistics of their data, resulting in a matrix of sensory distances. In general, embedding these sensory distances directly in a 2- or 3- dimensional Euclidean reconstruction is not possible because the sensory distance matrix may very well be non-Euclidean. However, standard *multidimensional scaling* (MDS) algorithms can be used to find an embedding which conforms as closely as possible to the specified distance matrix.

We adopted our basic assumption from [13]: that physically close sensors will tend to be more systematically related in their response statistics than physically distant sensors. This allowed us to estimate sensory distance either by using a direct information distance metric [24] as in [13], or by applying a more general metric construction algorithm to a statistical similarity measure (in the case of the present study, either linear correlation or mutual information). We briefly remark that the use of mutual information provides a fully non-linear comparison of sensor data streams; thus, the similarity between streams need not directly reflect spatial closeness, but may in fact reflect a more general type of statistical similarity [12], [13]. In the present tactile scenario this does not tend to be an issue because of the homogeneity of the sensors and the relative short range of the spatial stimuli. An exception was provided by the locomotion scenario for the cylinder (Fig. 4) which is governed by a more intricate relationship between physical proximity and statistical sensor similarity for the cylindrical object: contact between the cylinder and the ground tends to occur along a single axial line on the cylinder's surface.

The overall process was as follows:

- Begin with a number of tactile sensors arranged in some pattern over the surface of a rigid body, either in simulation or in hardware. Place the body in an environment which provides tactile stimulation of the sensors. (Simulations and physical experiments are described in more detail in later sections.)
- Record the data from all sensors over an appropriate number of time steps. (Currently, we consider only the pairwise instantaneous statistics of the data; temporal structure is ignored.)
- Generate a sensory distance matrix using each of the following candidate measures (described in more detail in later sections):
  1. Information Distance (as in [13])
  2. ISOMAP (based on information distance)
  3. ANISOMAP (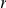-ANISOMAP using linear correlation and 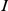-ANISOMAP using mutual information)
- Run the SMACOF [25] MDS algorithm (initialised as per standard with Torgerson scaling) on each generated distance matrix to create a reconstruction embedded in 3-dimensional space (for the simulations) or 2-dimensional and 3-dimensional space (for the hardware).

### Information Theory

This section briefly recaps some relevant definitions from information theory, which will be necessary to explain the information distance and 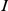-ANISOMAP reconstruction methods.

Mutual information 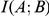 is a statistical measure which quantifies the amount of informational overlap between two random variables 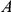 and 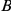.
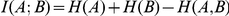
where 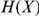 is the Shannon entropy of the random variable 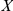, as follows, where 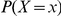 denotes the probability that the random variable 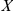 takes the value 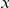, and 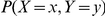 is the probability that random variables 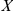 and 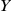, measured together, take the values 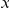 and 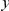 respectively. For empirically measured variables, 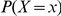 is taken to be the observed proportion of instances in which variable 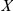 has the value 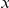, and similarly for 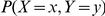.
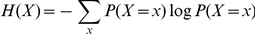


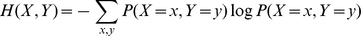
Note that 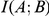 is not a normalised quantity; however, by standard results, it is guaranteed to fulfil the inequality 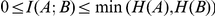 and is thus bounded by the capacity of the sensors.

Information-theoretic measures are amongst the most general measures of statistical relatedness, since in principle they capture all systematic relations, and not just linear ones. However, they are more difficult to measure than the linear correlation coefficient, both in terms of computational efficiency and in terms of reliably estimating them from small samples. In our experiments the sensors were designed to be identical, giving some advantage to the simpler non-information-theoretic linear correlation measure.

### Estimating Sensory Distance: Information Distance

For comparison with previous research, we considered the information distance measure 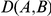 between two random variables 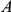 and 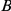 as used in [13]. The information distance (a metric; see [24]) is defined as
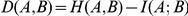
where 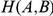 is the Shannon entropy of the joint distribution 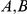 and 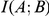 is the mutual information, as defined in the previous section.

Readings from each sensor were discretised into 4 bins (6 and 8 bin discretisations were also tested, and produced qualitatively similar results); this allowed empirical information-theoretic measures to be calculated exactly. The 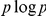 sums described in the previous section are computed exhaustively over every possible discrete outcome. The bin sizes were allocated using a maximum entropy binning following [26].

### Estimating Sensory Distance: ISOMAP

The ISOMAP algorithm, as described in [15], is a simple algorithm intended to reconstruct the structure of data points on a low-dimensional manifold embedded in a higher-dimensional space. This involves constructing a graph over the data in which only neighbouring points (chosen according to some pre-decided scheme: either by considering the 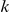 closest Euclidean neighbours of each data point, or all neighbours within a fixed distance 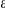) are directly connected by edges. The distance between non-neighbouring points is interpreted as the *shortest path length* within this graph.

One should note that the ISOMAP method was designed to preserve only the local structure of the data set. In certain contexts this may be desirable, but it does involve a loss of information about the more global relationships between reconstructed points.

For these experiments, we constructed 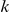-nearest-neighbour ISOMAP graphs as follows. Starting with an initially unconnected graph, we considered each sensor 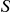 in turn and connected it to the 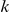 sensors which had the highest mutual information with 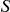. The ISOMAP distance matrix was then simply the matrix of shortest path lengths between sensors. The parameter 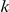 was chosen as the smallest such value yielding a totally connected graph.

### Estimating Sensory Distance: ANISOMAP

The ANISOMAP algorithm was developed to produce consistent distance estimates (i.e. a metric) between arbitrary objects, based on an initial matrix of dissimilarity values between objects. We will begin by describing this algorithm at a very general level, and then describe its application to sensoritopic reconstruction.

ANISOMAP operates in a similar graph-geodesic spirit to the ISOMAP algorithm [15], but with two key differences. Firstly, rather than considering only neighbouring points, *all* pairs of points are considered in ANISOMAP. This provides us with an increase in accuracy for the reconstruction of the long-range topological properties of the tactile map. Secondly, whereas ISOMAP initialises the edges of its graph with the (metric) distances between neighbouring points in some previously known space, ANISOMAP initialises the edges of its graph with some appropriate *semi-metric* (i.e. symmetric, zero diagonal, and elsewhere positive) matrix. This provides us with the flexibility of characterising spatial relatedness using originally non-spatial similarity measures.

For this, note that any semi-metric matrix 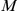 can be converted into a metric 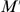 (“metricised”) in a natural way. First, we construct a totally-connected weighted graph 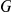, in which we identify the vertices 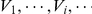 with the the rows of 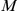 (or the columns, since 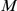 is symmetric). We identify the edge weights 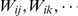 in 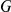 with the entries 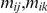 of 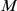. The metric 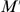 is then simply the matrix of *shortest path lengths* between pairs of vertices in 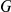. Note that if 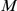 is itself a metric, then the metricised matrix 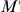 is identical to 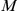 (see Text S3 for proof).

In the case of sensoritopic reconstruction, the statistical similarity between sensors provides more information about their relative position than their dissimilarity (since sensors at more or less any distance can be unrelated). In other words, shorter distance estimates are more reliable than longer ones.

Therefore, ANISOMAP uses the *shortest available consistent distance estimate*. This strikes a balance between local and global structure, in that when a shorter chain of estimated distances doesn't exist, the longer “dissimilarity” estimates are preserved intact, but when such a chain exists, it overrides the longer distance by virtue of the hypothesis of being more reliable.

In detail, the steps of the ANISOMAP algorithm are as follows:

1. Initialise a semi-metric matrix 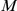 of direct dissimilarities between objects.
2. Construct a metric matrix 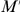 by running the Floyd-Warshall shortest-path algorithm [27], [28] with 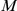 as input. (This algorithm computes the shortest paths between all vertices in a graph).
3. Embed 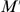 in the desired embedding space (e.g. 3D Euclidean space) using a suitable algorithm (e.g. 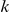-dimensional Euclidean space using SMACOF [25]).

If desired, we can allow the entries of 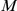 and 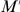 to take the value 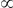 (technically making 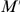 an *extended metric* rather than a metric).

It should be noted that this algorithm assumes a desired embedding space (e.g. 3D Euclidean space). An approach similar to that described in [6] could in principle be used to automatically choose a suitable dimensionality for the embedding space, but since our our focus here is on tactile robotics, in this study we simply assume a 3-dimensional target space from the outset.

This concludes the general description of the ANISOMAP algorithm. The next section will describe how we used it in sensoritopic reconstruction.

### 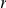-ANISOMAP and 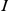-ANISOMAP

The ANISOMAP algorithm involves initialising a dissimilarity matrix 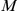 between pairs of sensors 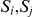 (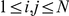), then generating a metric from 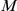 using a shortest-path measure. 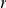-ANISOMAP and 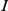-ANISOMAP were initialised using dissimilarity matrices 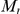 (derived from linear correlations between sensors) and 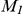 (derived from mutual informations between sensors) respectively. Each of these 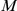
s were calculated by taking the reciprocal of some statistical similarity measure: the linear correlation coefficient 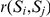 between sensor readings for 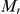, and the empirical mutual information 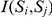 between sensor readings for 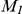. Since all sensors had the same characteristics, in the case of using 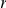, we set negative linear correlation values to zero. A small term proportional to the value of the smallest positive element 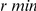 of the similarity matrix was added to the denominator of the fraction:
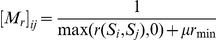


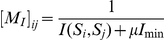
Note that this additive step is not strictly essential to the ANISOMAP algorithm, and instead 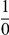 can be treated as equal to 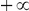. However, if this is done, then in general the distance matrix input to the multi-dimensional scaling algorithm for embedding into Euclidean space may contain infinities (i.e. the reconstructed points may be partitioned into disconnected sets). To avoid adapting the SMACOF scaling algorithm to separately have to handle this scenario, we decided to apply the above regularisation of infinite distances with a small additive term.

### Tactile Data: Simulated Environment

To provide physically relevant data for the 3D sensoritopic mapping algorithm, a number of simulations were run using the ODE physics engine [29]. Runs of 2000 simulation steps were performed for each of four different geometries in a “bombardment” scenario, and each of two different geometries in a “locomotion” scenario.

A summary of the experiments is given below, and the technical details of the simulations are provided in Text S1. Sample simulation runs for the various geometries and scenarios are provided as raw data in Data S1, S2, S3, S4, S5, S6.

#### Sensor Modelling

Sensors were modelled as short-range proximity sensors distributed on the surface of a rigid object. The sensors contributed nothing to the dynamics of the object, and only registered a non-zero signal when a simulated detectable body came within the sensor's range. The sensor's signal was proportional to the detectable body's maximum penetration into the sensor's sphere of detection.

This tactile simulation was similar in spirit to the “simulated baby” in [10], although our approach is simpler and focuses on the explicit reconstruction of tactile surfaces.

#### Geometries

**Figure 5:**
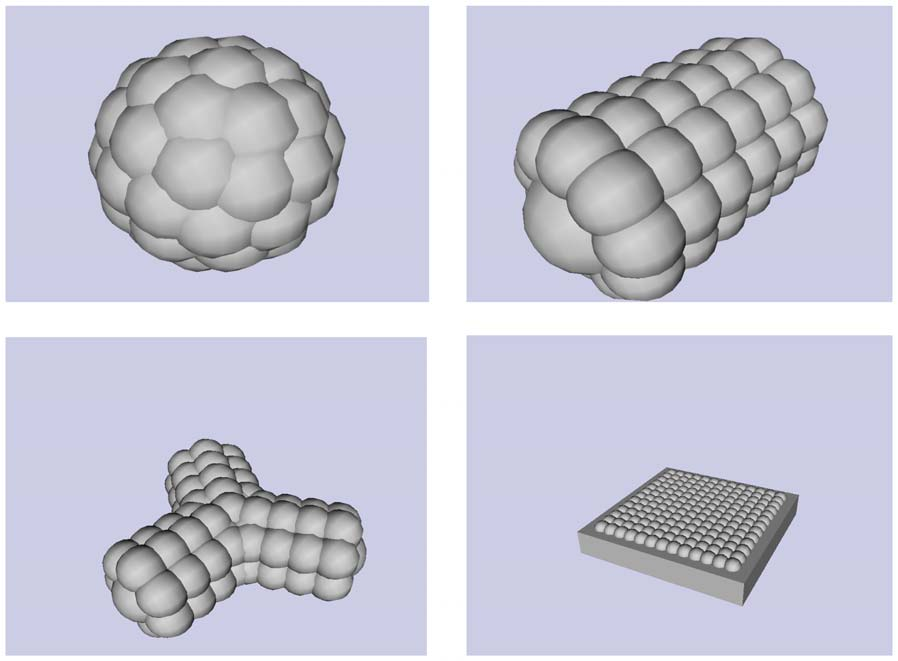
Simulated sensor geometries. The sphere (60 sensors), cylinder (60 sensors, none on the ends), y-shape (105 sensors), and plane (169 sensors) geometries.

Four topologies were simulated: 60 sensors distributed uniformly on the surface of a sphere, 60 sensors distributed in 10 columns of 6 on the surface of a cylindrical object, 105 sensors distributed on the surface of a compound y-shape object, and 169 sensors distributed in a grid of 13 by 13 on one surface of a cuboid (see Fig. 5).

#### Scenarios

**Figure 6:**
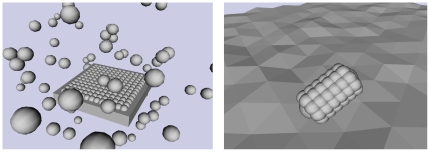
Simulated scenarios. Bombardment and locomotion.

Two different physical scenarios were simulated:

- *Bombardment*. The sensor object (sphere, cylinder, y-shape or plane) was fixed in the centre of a zero-gravity box filled with detectable bodies in the form of moving elastic balls. The object's sensors detected collisions between the balls and the sensors' sphere of sensitivity (the balls also collided with each other and with the walls of the box). See Fig. 6.
- *Locomotion*. The sensor object (sphere or cylinder) was propelled along uneven detectable terrain under simulated gravity. The object's sensors detected contact with the simulated terrain within their spheres of sensitivity. See Fig. 6.

### Tactile Data: Physical Environment

We conducted some experiments using a first release hardware prototype constructed by the Italian Institute of Technology (IIT) [30] as part of the RoboSKIN project. RoboSKIN is an EU project which aims to develop artificial skin hardware, middleware and software technologies in the context of autonomous robotics. The skin prototype consisted of a hexagonal patch of 72 capacitative sensors, mounted on a flexible substrate and arranged in 6 triangles of 12 sensors each. The entire patch is covered with a layer of rubbery silicone. (See Figure S10 in supporting information for illustration.)

#### Prototyping Issues

For reasons to do with the prototype hardware driver, the readings for an entire triangle (12 sensors) on the prototype was always zero, so we ignored these sensors (we coded a simple preprocessing step which removes from the dataset any sensors whose values do not vary). This missing triangle provided an additional opportunity to gauge the reconstruction capability of our algorithm.

Additionally, a single capacitative sensor (labelled ‘30’ in Fig. S10) suffered hardware failure between experimental runs. The sensor was functional during runs 1 to 3 and faulty in runs 4 and 5. This sensor had a tendency to saturate intermittently regardless of whether it was being physically stimulated or not; note that the readings from this sensor were included in the reconstruction data.

These prototyping issues with the hardware proved an advantage, by demonstrating that the ANISOMAP-based algorithm can in principle cope with several forms of real-world complexity: both non-uniform geometry (from the missing triangle) and unanticipated hardware failure (from the failed sensor). This is in spirit analogous to the results in [9], where a robot using an evolution-based self-modelling technique was able to adapt spontaneously to damage. The skin patch was stimulated by hand in an ad-hoc manner over a relatively short time period, so we show the hardware data here mainly to illustrate the method's potential.

**Figure 7:**
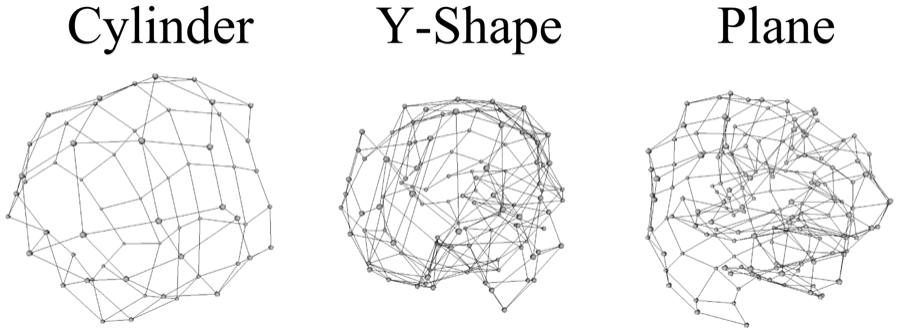
Typical information distance reconstructions for the simulation experiments, showing “spherisation”. Left: reconstructed cylinder; centre: reconstructed Y-shape; right: reconstructed plane.

It can be seen clearly in figure 7 (and in some of the reconstructions in the supporting information online, see Figures S1, S2, S3) that the information distance measure has a tendency to “spherise” reconstructions, spreading the reconstructed points around a 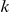-sphere. This phenomenon was discussed in one of our earlier papers [31]: to summarise the idea, most sensors are unrelated, and have an information distance which is approximately equal to the sum of the entropies of the individual sensors (each of which is approximately equal). These near-identical distances between unrelated sensors cannot be reproduced in a low-dimensional Euclidean reconstruction, with the consequence that the information-distance-based reconstructions tend to “bulge”. In two dimensions, the effect is less dominant than in three; this can be seen most clearly in figure 3, where three-dimensional reconstructions using information distance are notably worse than two-dimensional ones (this is not true for the other reconstruction methods).

#### Experimental Data

In order to validate the reconstruction algorithm in hardware, we stimulated the prototype skin patch by hand with an irregular rubbing motion. This stimulation involved circular and linear motions in various directions pressing the palm of the hand against the skin patch. Pressure values from 60 sensors on the 5 responsive triangles were recorded over time and used as inputs for our reconstruction algorithms.

Five runs were recorded of respectively 1120, 1054, 1487, 2128 and 2105 time steps each. The raw data for the runs is provided in supporting information Data S7, S8, S9, S10, S11, with the data format described in Text S2.

## Supporting Information

Figure S1 **Randomly selected reconstructions for the cylinder (bombardment scenario) for all reconstruction algorithms.**
(TIF)

Figure S2 **Randomly selected reconstructions for the plane (bombardment scenario) for all reconstruction algorithms.**
(TIF)

Figure S3 **Randomly selected reconstructions for the Y-shape (bombardment scenario) for all reconstruction algorithms.**
(TIF)

Figure S4 **Randomly selected reconstructions for the sphere (bombardment scenario) for all reconstruction algorithms.**
(TIF)

Figure S5 **All reconstructions for hardware experiment 1.**
(TIF)

Figure S6 **All reconstructions for hardware experiment 2.**
(TIF)

Figure S7 **All reconstructions for hardware experiment 3.**
(TIF)

Figure S8 **All reconstructions for hardware experiment 4.**
(TIF)

Figure S9 **All reconstructions for hardware experiment 5.**
(TIF)

Figure S10 **Hardware patch schematic.**
(TIF)

Text S1 **Summary of simulation method.**
(TXT)

Text S2 **Summary of format for hardware data.**
(TXT)

Text S3 **Proof: shortest-path metricisation leaves a metric matrix unaltered.**
(PDF)

Data S1 **Sample simulation run output (cylinder bombardment).**
(TXT)

Data S2 **Sample simulation run output (sphere bombardment).**
(TXT)

Data S3 **Sample simulation run output (plane bombardment).**
(TXT)

Data S4 **Sample simulation run output (yshape bombardment).**
(TXT)

Data S5 **Sample simulation run output (cylinder locomotion).**
(TXT)

Data S6 **Sample simulation run output (sphere locomotion).**
(TXT)

Data S7 **Hardware experiment data (experiment 1).**
(LOG)

Data S8 **Hardware experiment data (experiment 2).**
(LOG)

Data S9 **Hardware experiment data (experiment 3).**
(LOG)

Data S10 **Hardware experiment data (experiment 4).**
(LOG)

Data S11 **Hardware experiment data (experiment 5).**
(LOG)
