# Supplementary figures and images for: Generation of Tactile Maps for Artificial Skin
Source: PLoS One. 2011 Nov 10;6(11):e26561. doi: 10.1371/journal.pone.0026561 (PMC3213097; doi:10.1371/journal.pone.0026561)

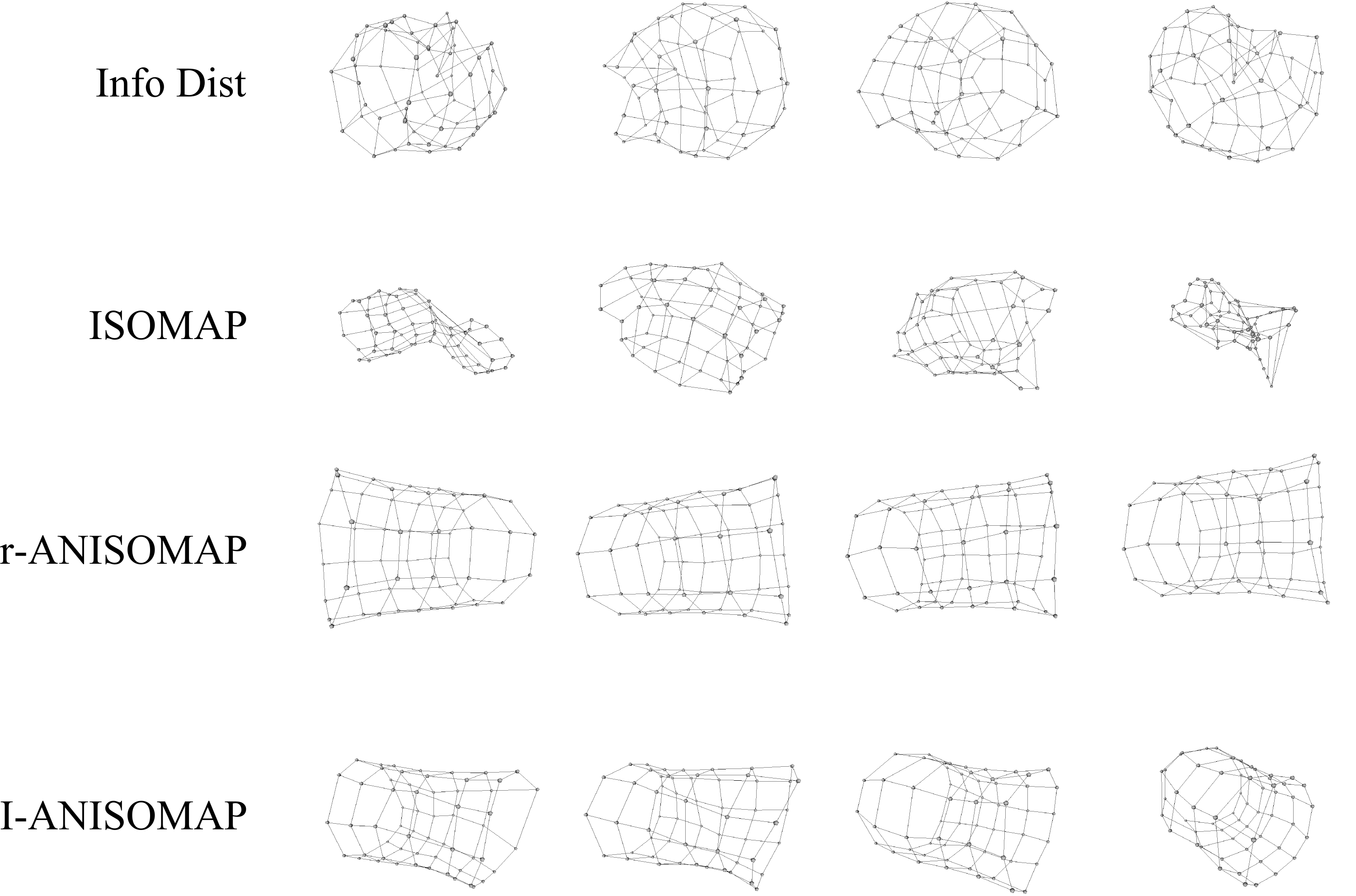

Supplement: Figure S1 — Randomly selected reconstructions for the cylinder (bombardment scenario) for all reconstruction algorithms. (TIF) [file pone.0026561.s001.tif]

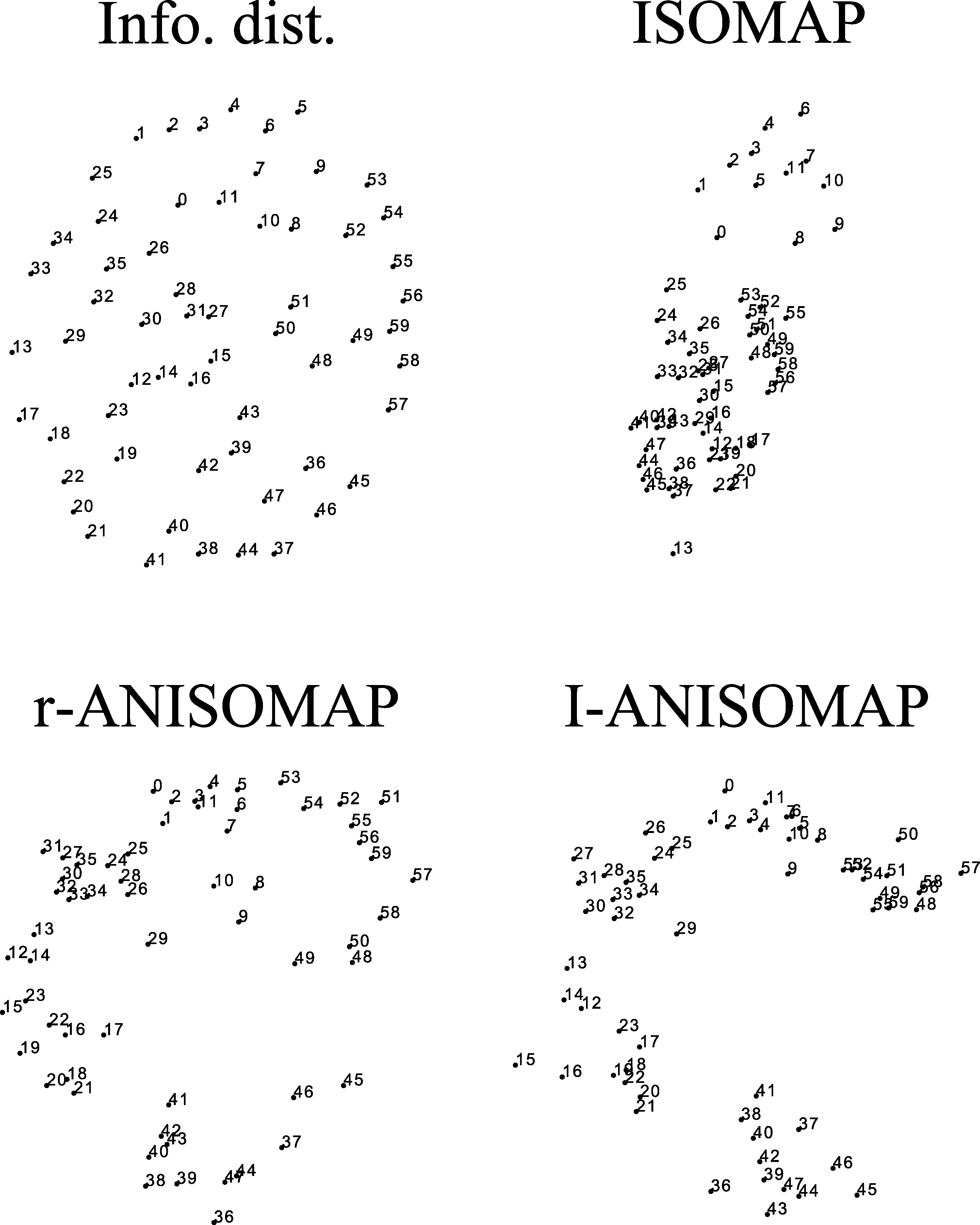

Supplement: Figure S5 — All reconstructions for hardware experiment 1. (TIF) [file pone.0026561.s005.tif]

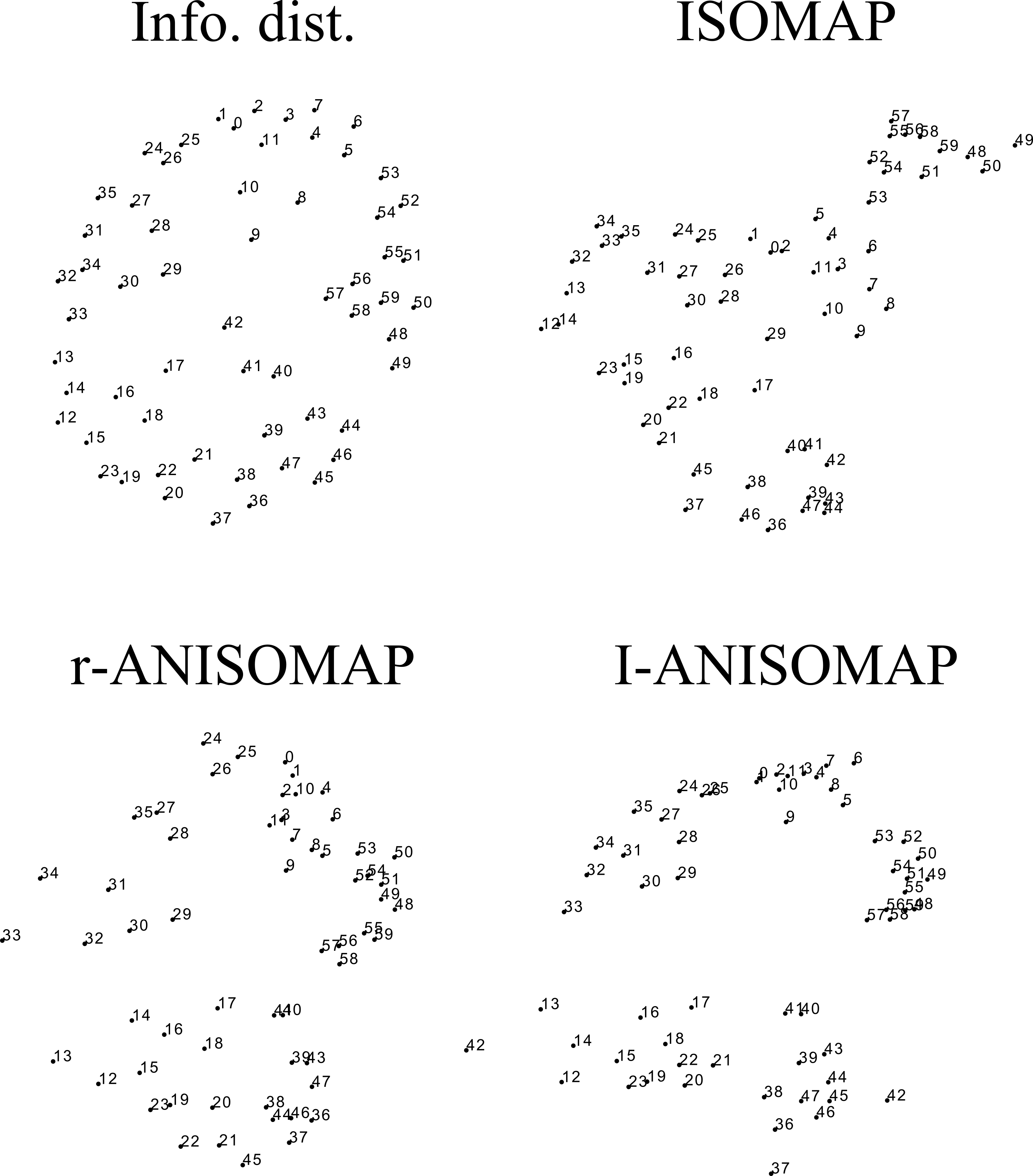

Supplement: Figure S6 — All reconstructions for hardware experiment 2. (TIF) [file pone.0026561.s006.tif]

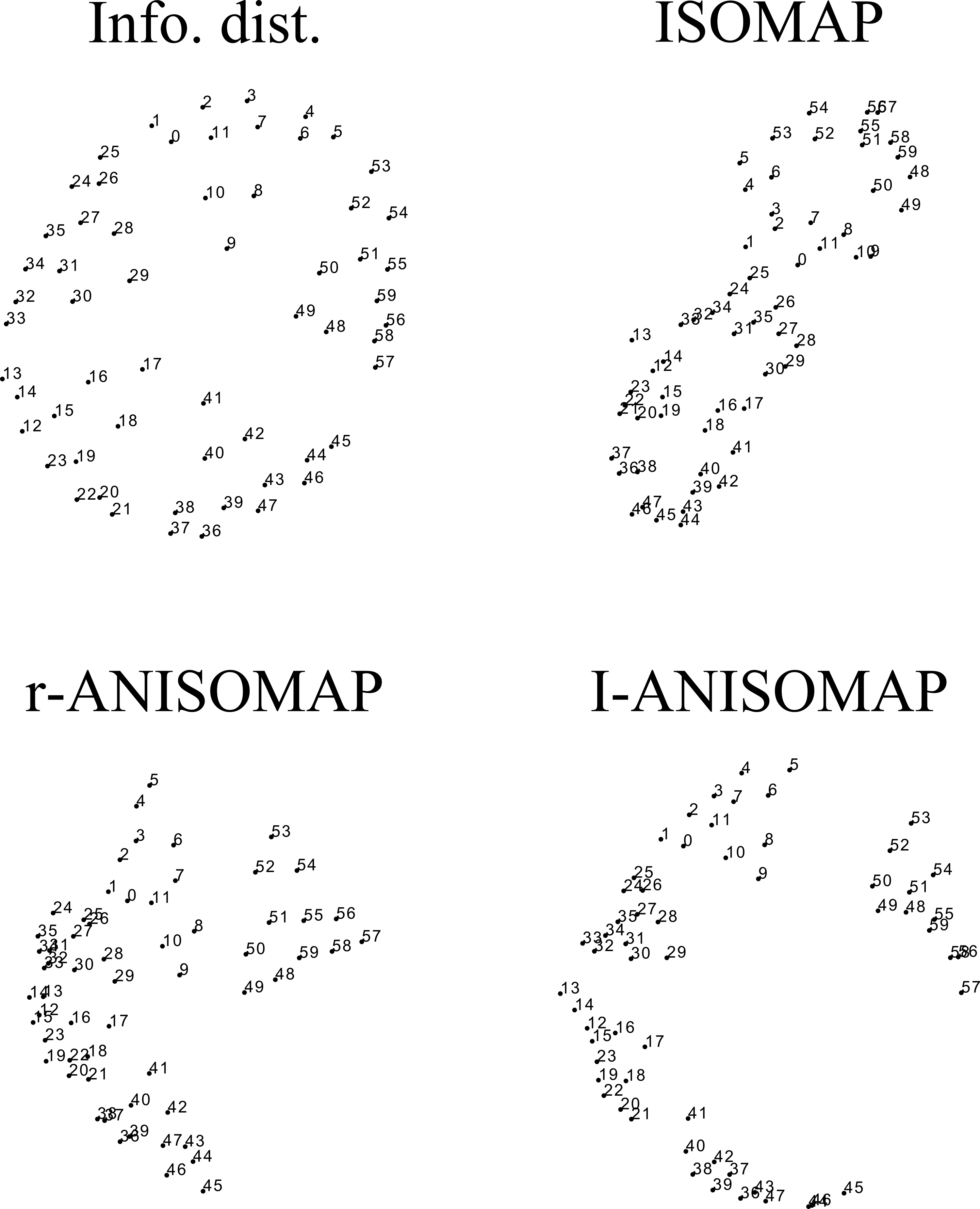

Supplement: Figure S7 — All reconstructions for hardware experiment 3. (TIF) [file pone.0026561.s007.tif]

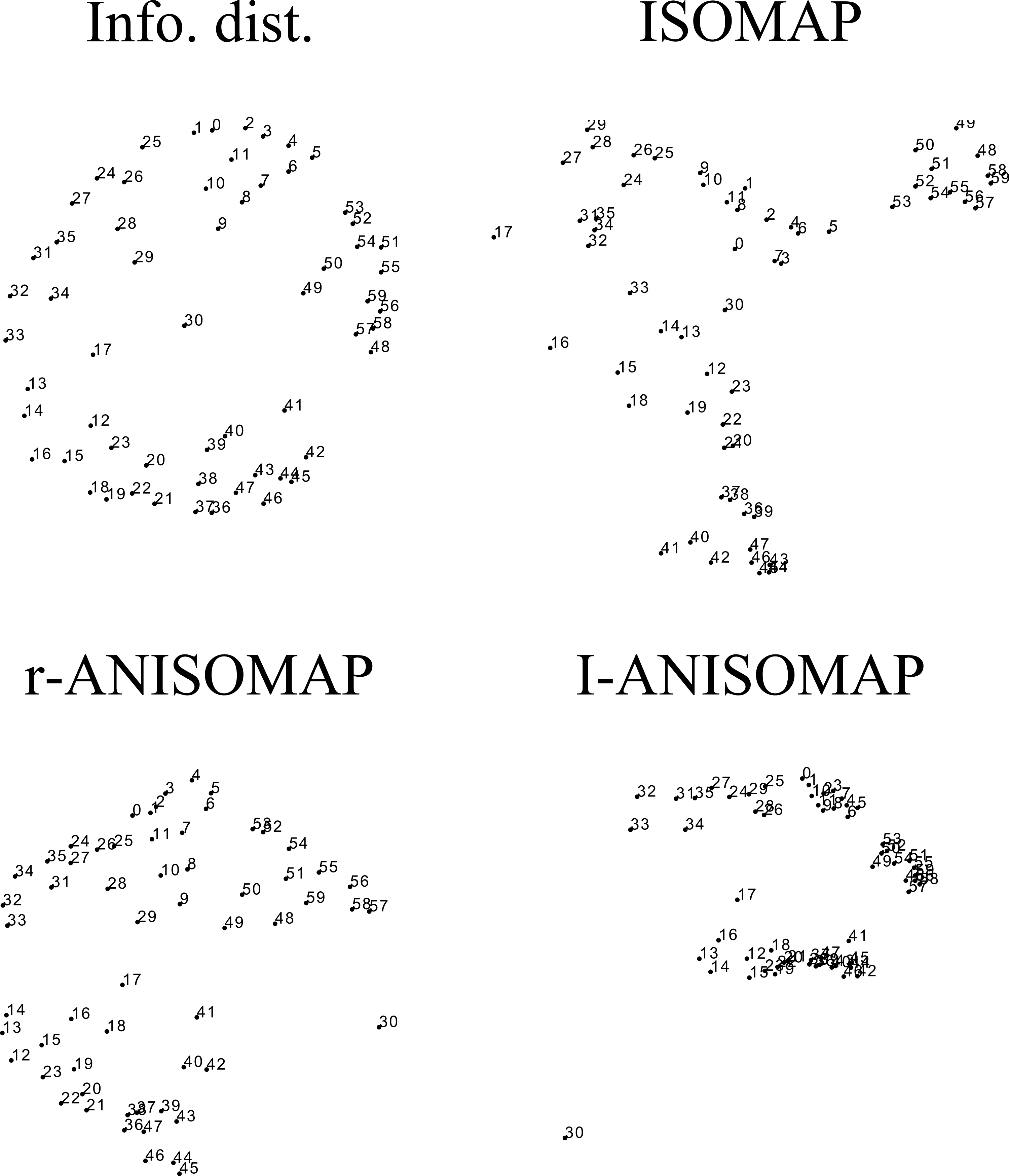

Supplement: Figure S8 — All reconstructions for hardware experiment 4. (TIF) [file pone.0026561.s008.tif]

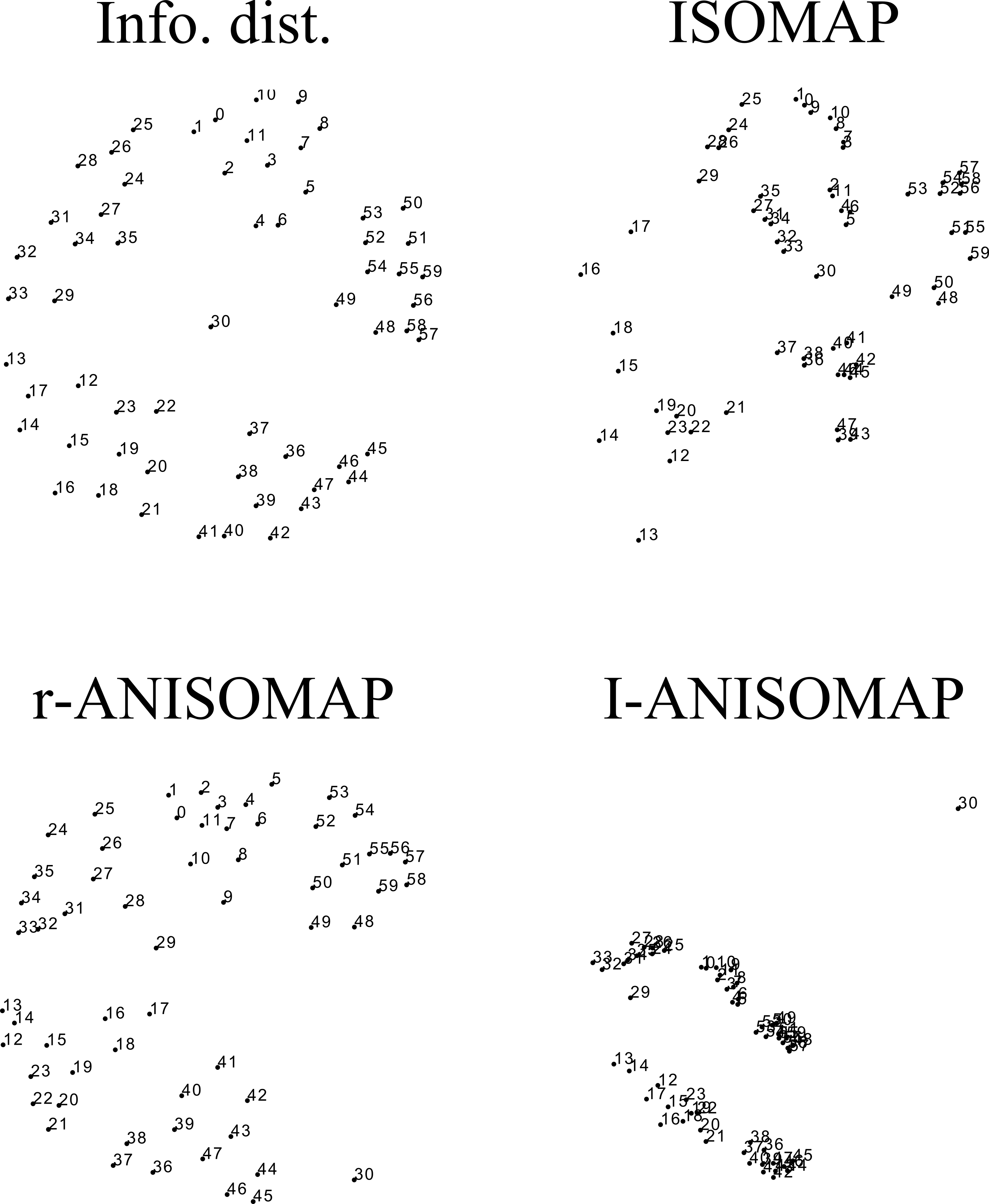

Supplement: Figure S9 — All reconstructions for hardware experiment 5. (TIF) [file pone.0026561.s009.tif]

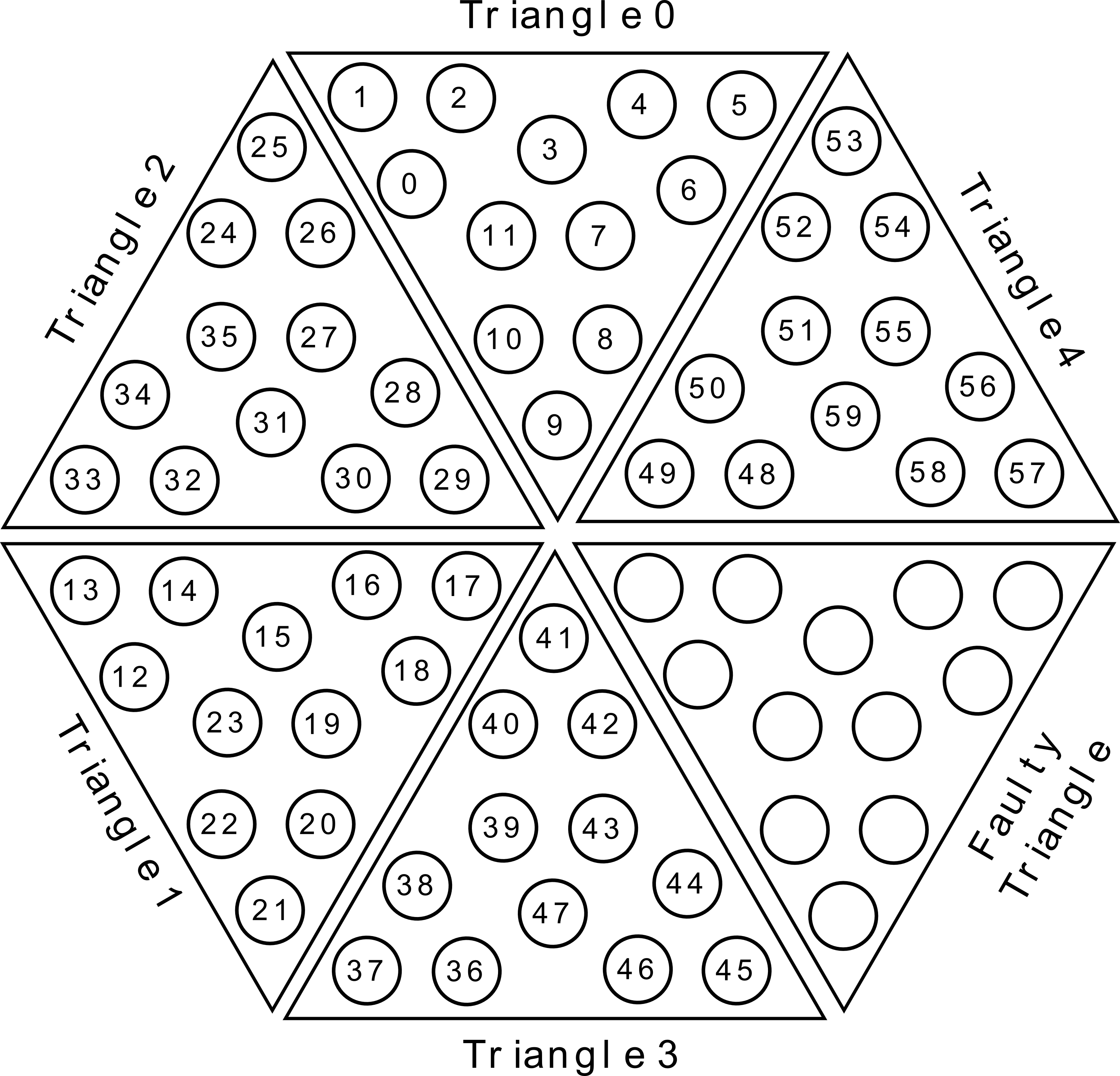

Supplement: Figure S10 — Hardware patch schematic. (TIF) [file pone.0026561.s010.tif]
