# Supplementary material for: Generation of Tactile Maps for Artificial Skin
Source: PLoS One. 2011 Nov 10;6(11):e26561. doi: 10.1371/journal.pone.0026561 (PMC3213097; doi:10.1371/journal.pone.0026561)
Supplement: Text S3 — Proof: shortest-path metricisation leaves a metric matrix unaltered. (PDF) [file pone.0026561.s013.pdf]

Proof: metric matrices are unchanged by shortest-path metricisation

Consider a finite fully-connected (undirected) weighted graph G with vertices $V_1, \cdots, V_n \in G$. Define $E(V_i, V_j)$ as the weight associated with the edge connecting V_i and V_j. If E is a metric, then the shortest path length between any two vertices V_i, V_j is equal to E (i.e. the direct link is the shortest path).

The proof proceeds by contradiction. Assume that there are some pairs of vertices $V_i, V_j \in G$ with shortest path lengths $L(V_i, V_j)$ which are different from their directly connecting edge weights $E(V_i, V_j)$.

Pick some (not necessarily unique) pair V_a, V_b with the smallest such shortest path length, i.e. with

$$L(V_a, V_b) \neq E(V_a, V_b)$$

and

$$\forall\, V_i, V_j \in G : L(V_i, V_j) < L(V_a, V_b) \implies L(V_i, V_j) = E(V_i, V_j)$$

By definition of "shortest path", $L(V_a, V_b) \leq E(V_a, V_b)$, so $L(V_a, V_b) < E(V_a, V_b)$.

Then $L(V_a, V_b) = E(V_a, V_c) + L(V_c, V_b)$ for some $c \notin \{a, b\}$ (again, by definition of "shortest path"). But $E(V_a, V_c) > 0$ (metric axiom), so $L(V_c, V_b) < L(V_a, V_b)$.

Because of how we chose V_a, V_b, this implies that $L(V_c, V_b) = E(V_c, V_b)$. Hence $L(V_a, V_c) = E(V_a, V_b) + E(V_c, V_b) < E(V_a, V_b)$, which violates the triangle inequality metric axiom for E and concludes the proof.
